# Supplementary material for: Effects of Taekwondo intervention on balance ability: A meta-analysis and systematic review
Source: PLoS One. 2025 Feb 13;20(2):e0317844. doi: 10.1371/journal.pone.0317844 (PMC11825010; doi:10.1371/journal.pone.0317844)
Supplement: S1 File — (DOCX) [file pone.0317844.s003.docx]

**Search Strategy**

We employed a search strategy using "Keyword 1 + Keyword 2," which included both the full terms and abbreviations for all relevant terms. Keyword 1 was set as "Taekwondo," and Keyword 2 was set as "Balance" (in Chinese, this includes "balance control ability" and "balance ability"). To maximize the retrieval of all relevant literature, the search term was set as "Balance." During the search, documents other than dissertations and journal articles (such as conference papers, patents, newspapers, etc.) were excluded

The specific search strategy is as follows:

**Chinese Databases**

CNKI

(主题：跆拳道（精确））AND（主题：平衡（精确））🡪(Topic:Taekwondo(Accurate))AND(Topic:Balance(Accurate))

WANFANG DATA

#主题:("跆拳道") and 主题:("平衡")🡪#Topic:(" Taekwondo ") and Topic:(" Balance")

**Korean Databases 302**

RISS

#논문명 : 태권도 <AND> 논문명 : 균형🡪#Title: Taekwondo<AND> Title: Balance

#논문명 : 태권도 <AND> 주제어 : 균형🡪#Title: Taekwondo<AND> Topic: Balance

#논문명 : 태권도 <AND> 초록 : 균형🡪#Title: Taekwondo<AND> Abstract: Balance

KISS

#논문명 : 태권도 <AND> 논문명 : 균형🡪#Title: Taekwondo<AND> Title: Balance

DBPIA

#논문명 : 태권도 <AND> 논문명 : 균형🡪#Title: Taekwondo<AND> Title: Balance

**English Databases**

Web of Science

# Topic: Taekwondo<AND> Topic: Balance

PubMed

# Topic: Taekwondo<AND> Topic: Balance
