# Supplementary material for: Effects of Taekwondo intervention on balance ability: A meta-analysis and systematic review
Source: PLoS One. 2025 Feb 13;20(2):e0317844. doi: 10.1371/journal.pone.0317844 (PMC11825010; doi:10.1371/journal.pone.0317844)
Supplement: S2 File — (DOCX) [file pone.0317844.s004.docx]

**Description of Balance Assessment Methods**

The following is a brief introduction to the methods used in this study to assess balance ability. Please note that these are not standardized procedures but rather general processes that may be adjusted according to the specific research objectives.

**1. Single-Leg Stance with Eyes Closed**

**Procedure:**

The participant stands on a flat surface with arms at their sides.

With eyes closed, they lift one leg while the other leg remains in support.

Time is recorded from the moment the foot is lifted until balance is lost or the foot touches the ground.

**Purpose:**

Assesses static balance ability without visual feedback.

Longer times indicate better balance.

**2. Marching in Place with Eyes Closed**

**Procedure:**

The participant stands on a flat surface with their feet placed within a circular area with a 20 cm radius, marked on the ground.

After closing their eyes, they are instructed to march in place by lifting their knees alternately at a consistent pace.

Time is recorded from the start of marching until one foot steps out of the circular area or touches the boundary line.

The longer the duration, the better the dynamic balance ability.

**Purpose:**

Evaluates dynamic balance and proprioceptive control under conditions without visual feedback.

**3. Y Balance Test**

**Procedure:**

The participant stands at the center of a Y-shaped grid, balancing on one leg while the other leg reaches in specific directions.

The participant reaches as far as possible in three directions: anterior, posteromedial, and posterolateral.

The maximum reach distance in each direction is recorded.

**Purpose:**

Tests lower-limb dynamic balance and movement control.

**4. Timed Up-and-Go Test**

**Procedure:**

The participant starts seated in a standard chair with knees bent at a 90-degree angle.

On the command, they stand up, walk 3 meters, turn around, walk back, and sit down again.

Time is recorded from the moment they stand up until they return to a seated position.

Purpose:

Measures dynamic balance and functional mobility.

Faster times indicate better functional movement.

**5. Functional Reach Test**

**Procedure:**

The participant stands on a flat surface with feet shoulder-width apart.

With one arm extended forward, the starting position of the fingertips is recorded.

Without moving their feet, the participant reaches forward as far as possible, and the endpoint of the fingertips is recorded.

The average of two trials is calculated.

**Purpose:**

Assesses anterior dynamic balance and reach ability.

Longer reach distances indicate better balance.
