# Supplementary material for: Effects of Taekwondo intervention on balance ability: A meta-analysis and systematic review
Source: PLoS One. 2025 Feb 13;20(2):e0317844. doi: 10.1371/journal.pone.0317844 (PMC11825010; doi:10.1371/journal.pone.0317844)
Supplement: Attachment — Submitted filename: Review for research paper_PONE-D-24-37293.pdf [file pone.0317844.s006.pdf]

Review for research paper "Effects of Taekwondo Intervention on Balance Ability: A Meta-Analysis and Systematic Review" by Han Zhengfa and Ju Hanyu

Dear Editor,

The paper "Effects of Taekwondo Intervention on Balance Ability: A Meta-Analysis and Systematic Review" presents a comprehensive meta-analysis and systematic review of the effects of Taekwondo interventions on balance ability. The study is well-structured, with a clear objective, detailed methodology, and thorough quantitative and qualitative data analysis. The findings indicate that the data supports the proposed optimal intervention protocol of 60-70 minutes per session, once weekly for 12 weeks.

The paper is well-structured and comprehensive, but a few areas could be improved. I recommend publishing the manuscript "Effects of Taekwondo Intervention on Balance Ability: A Meta-Analysis and Systematic Review" after a major review. The following issues should be improved or clarified (the pages were numbered starting from the manuscript's page 9):

1. P 9. Line 27: To add the results of bias for studies. Was it low or high?
2. P 10. L 33. What kind of training do you mean in this sentence: *for and a times length of 60-70 minutes*? Taekwondo or balance training?
3. P 12. L 60. What kind of intervention? Explain in more detail.
4. P 12. L 73. Specify what PRISMA stands for.
5. P 13. L 79. Add the starting date as well.
6. P 13. L 90. Write the full name for *PICOS.*
7. P 13. L 94. My suggestion is, instead of *Taekwondo Gymnastic*, to write Freestyle taekwondo.
8. P 14. L 106. Which version? Write a proper citation.
9. P 14. L 113. Reference it.
10. P 15. L 129 and 130 are redundant and previously repeated. Delete them.
11. P 16. L 157. Explain briefly the moderate intensity of 7 studies. Were they based on heart rate or other factors?
12. P 17. L 159. Explain the test *"single-leg stance with eyes closed."*
13. P 18 and 19. In Table 1, *the Period section* specifies whether it is a Week or a Month.
14. P 19. For Pons Van Dijk's (2013) reference, it is unclear whether there are 40 sessions per month or week.
15. P 19. Correct the size *of 60 min in Table 1 for Kim (2024).*
16. P 21. Do you mean **there are** in this sentence? I think it's a typo mistake. *Regarding overall bias, the ame four studies.*
17. P21. Write RTCs instead of *rcts* for both Fig. 2 and Fig. 3.
18. P 24. Please write the statistical analysis summary in Figure 4.
19. P 24. In this sentence, *Visual inspection of the funnel plot,* you should write (Figure 5).
20. P 24. Where is the result of Egger's test?
21. P 24. Having the effect sizes per study name inside the funnel plot in Fig. 5 will be great—*funnel Plot.*
22. P 26. There is a misinterpretation of the results and p-value for *Table 5*. In the last paragraph of this page, you wrote: *Additionally, significant moderating effects were observed for Period, Times per week, and minutes per Time (p<0.05), but not for Sex and Age (p>0.05).* The p-values for gender in male and female groups and Ages 4-6 and 18-21 are significant.

23. P 27. You should change and write Table 6 in the text instead of Table 4 in this sentence: *for this method. The heterogeneity tests and meta-analysis results are presented in Table 4.*
24. P 27. All tables indicate the significant value for p. Is it $p<0.05$ or $p<0.01$?
25. P 28. You should change and write Table 7 in the text instead of Table 5 in this sentence: *presented in Table 5. The analysis revealed substantial heterogeneity for the "X speed (mm/s)."*
26. Table 7 shows the heterogeneity results for length (mm), which show $I^2 = 57.868$ and $p = 0.050$. Isn't heterogeneity marginally significant?
27. P 33. L 102 – 106, revise based on 22—p 26.
28. P 37. L 188. Which study? Write the reference number.
29. P 48. For Fig. 4, the overall estimated effect size and heterogeneity test results are needed.
