# Supplementary material for: Effects of Taekwondo intervention on balance ability: A meta-analysis and systematic review
Source: PLoS One. 2025 Feb 13;20(2):e0317844. doi: 10.1371/journal.pone.0317844 (PMC11825010; doi:10.1371/journal.pone.0317844)
Supplement: Attachment — Submitted filename: Response to Reviewer 2.doc [file pone.0317844.s007.doc]

**Dear Reviewer,**

Thank you for your valuable feedback and suggestions on our manuscript titled “[Effects of Taekwondo Intervention on Balance Ability: A Meta-Analysis and Systematic Review]”. We sincerely appreciate the time and effort you put into reviewing our work.

We have carefully considered all your comments and have made the necessary revisions to the manuscript accordingly. To facilitate your review, the modified sections have been highlighted in yellow within the revised manuscript.

Below, we provide detailed responses to each of your comments:

1. P 9. Line 27: To add the results of bias for studies. Was it low or high?

**Answer: We have added a description of the risk of bias test results in the results summary section. Line:27-29**

2. P 10. L 33. What kind of training do you mean in this sentence: for and a times length of 60-70 minutes? Taekwondo or balance training?

**Answer: This refers to Taekwondo training, and we have revised the text accordingly. Line:35**

3. P 12. L 60. What kind of intervention? Explain in more detail.

**Answer:** **This refers to general Taekwondo training. Typically, training for beginners includes basic elements of Taekwondo, such as fundamental footwork, punching techniques, and kicking techniques. We have modified the relevant descriptions in the manuscript. Line:64-68**

4. P 12. L 73. Specify what PRISMA stands for.

**Answer: We have included the full name of PRISMA in the manuscript. Line:79-80**

5. P 13. L 79. Add the starting date as well.

**Answer: We have added the start date of the study to the manuscript. Line:86-87**

6. P 13. L 90. Write the full name for PICOS.

**Answer: We have included the full name of PICOS in the manuscript. Line:97-98**

7. P 13. L 94. My suggestion is, instead of Taekwondo Gymnastic, to write Freestyle taekwondo.

**Answer: Taekwondo Gymnastic refers to a form of training that combines basic Taekwondo movements with gymnastic elements, creating a rhythmic exercise routine. It is particularly suitable for beginners as it provides an accessible and engaging introduction to Taekwondo. On the other hand, Freestyle Taekwondo is a form of Taekwondo Poomsae (patterns) and is recognized as a competitive event in Taekwondo Poomsae competitions. Freestyle Taekwondo is generally not suitable for beginners due to its higher level of difficulty and is primarily practiced by professional athletes. Therefore, we believe that "Taekwondo Gymnastic" is a more appropriate term for the context of our study.**

8. P 14. L 106. Which version? Write a proper citation.

**Answer: We have specified the software version in the manuscript and added the corresponding reference to the reference list. Line:114**

9. P 14. L 113. Reference it.

**Answer: We have added the relevant references. Line:121**

10. P 15. L 129 and 130 are redundant and previously repeated. Delete them.

**Answer: We have removed the duplicated content.**

11. P 16. L 157. Explain briefly the moderate intensity of 7 studies. Were they based on heart rate or other factors?

**Answer: We have added the basis for intensity assessment in Table 1.**

12. P 17. L 159. Explain the test "single-leg stance with eyes closed."

**Answer: The content of the single-leg stance with eyes closed test is simply the duration of standing on one leg, which has been explained in Table 1. Additionally, we have submitted a supplementary document titled "Description of Balance Assessment Methods" as an additional information file. This document provides a brief introduction to the testing procedures and purposes of the balance assessment methods mentioned in this study.**

13. P 18 and 19. In Table 1, the Period section specifies whether it is a Week or a Month.

**Answer: Since most of the included studies reported the duration in weeks, but some used months as the unit, we have updated Table 1 to clearly indicate whether the duration is in weeks or months.**

14. P 19. For Pons Van Dijk's (2013) reference, it is unclear whether there are 40 sessions per month or week.

**Answer: Since this study is unique, with a total of 40 training sessions conducted over 15 months, we have marked it with an asterisk (*) and provided a note at the end of the table for clarification, to distinguish it from the others.**

15. P 19. Correct the size of 60 min in Table 1 for Kim (2024).

**Answer: It has been corrected.**

16. P 21. Do you mean there are in this sentence? I think it’s a typo mistake. Regarding overall bias, the ame four studies.

**Answer: It has been corrected. Line:186-187**

17. P21. Write RTCs instead of rcts for both Fig. 2 and Fig. 3.

**Answer: It has been corrected.**

18. P 24. Please write the statistical analysis summary in Figure 4.

**Answer: It has been added in Figure 4.**

19. P 24. In this sentence, Visual inspection of the funnel plot, you should write (Figure 5).

**Answer: It has been corrected. Line:250-251**

20. P 24. Where is the result of Egger's test?

**Answer: The results of Egger's test are described in the Publication Bias Assessment section. Additionally, we have included detailed results, such as Egger's test, in the funnel plot. Line:253-254**

21. P 24. Having the effect sizes per study name inside the funnel plot in Fig. 5 will be great—funnel Plot.

**Answer: We have marked the effect sizes of each study in the funnel plot.**

22. P 26. There is a misinterpretation of the results and p-value for Table 5. In the last paragraph of this page, you wrote: Additionally, significant moderating effects were observed for Period, Times per week, and minutes per Time (p<0.05), but not for Sex and Age (p>0.05). The p-values for gender in male and female groups and Ages 4-6 and 18-21 are significant.

**Answer: You may have misinterpreted this; the moderating effects of gender (p = 0.786) and age (p = 0.300) are not significant.**

23. P 27. You should change and write Table 6 in the text instead of Table 4 in this sentence: for this method. The heterogeneity tests and meta-analysis results are presented in Table 4.

**Answer: It has been corrected.**

24. P 27. All tables indicate the significant value for p. Is it p<0.05 or p<0.01?

**Answer: We have added a description of the significance threshold in the "2.5 Statistical Analysis" section. Line:150-151**

25. P 28. You should change and write Table 7 in the text instead of Table 5 in this sentence: presented in Table 5. The analysis revealed substantial heterogeneity for the "X speed (mm/s)."

**Answer: It has been corrected.**

26. Table 7 shows the heterogeneity results for length (mm), which show I2 = 57.868 and p =0.050. Isn't heterogeneity marginally significant?

**Answer: The p-value reported here is rounded to two decimal places. The actual p-value is 0.0501…, which is greater than 0.05. Therefore, the heterogeneity is not statistically significant.**

27. P 33. L 102 – 106, revise based on 22—p 26.

**Answer: The moderating effects of gender (p = 0.786) and age (p = 0.300) are not statistically significant.**

28. P 37. L 188. Which study? Write the reference number.

**Answer: This was an issue with our language expression, and it has been revised. Line:505-511**

29. P 48. For Fig. 4, the overall estimated effect size and heterogeneity test results are needed.

**Answer: We have added information on the overall effect size and heterogeneity results to Figure 4.**

We hope the revised manuscript addresses all your concerns adequately. Please feel free to let us know if there are any additional suggestions or further modifications required.

Thank you again for your valuable input, which has greatly improved the quality of our work.

Sincerely,
